# Supplementary material for: Reduced visual context effects in global motion processing in depression
Source: PLoS One. 2023 Sep 13;18(9):e0291513. doi: 10.1371/journal.pone.0291513 (PMC10499266; doi:10.1371/journal.pone.0291513)
Supplement: S1 Text — (DOCX) [file pone.0291513.s001.docx]

# Supporting information

Due to the differences in the results that were obtained when medicated participants were or were not included in the main analyses, the following supplementary analyses are included to clarify differences in accuracy and contextual effects by medication status within the depressed group.

## Medicated vs. unmedicated participants

In the following analyses, participants within the depressed group (remitted and current) were compared by medication status (currently taking any psychotropic medication vs. currently taking no psychotropic medications). There was no main effect of group for baseline accuracy (F(1, 44)=0.207, p=0.65, $\eta^{2}=0.005, 95\% \eta^{2}\mathrm{CI}[0.00, 0.08]$) and there was no significant medication status by coherence interaction (F(3.28, 144.36)=0.82, p=0.494, $\eta^{2}=0.018, 95\% \eta^{2}\mathrm{CI}[0.00, 0.048]$), but there was a significant main effect of coherence (F(3.28,144.36)=82.33, p<.001, $\eta^{2}=0.652, 95\% \eta^{2}\mathrm{CI}[0.57, 0.70]$; Supplementary Figure 1). A three-way ANOVA compared accuracy levels by medication status, direction, and coherence (Supplementary Figure 2-A). There were significant main effects of direction (F(1,44)=60.20, p<.001, $\eta^{2}=0.578, 95\% \eta^{2}\mathrm{CI}[0.405, 0.679]$) and coherence (F(2.977,130.993)=52.267, p<.001, $\eta^{2}=0.543, 95\% \eta^{2}\mathrm{CI}[0.440, 0.708]$). There was a significant direction by medication status interaction (F(1,44)=9.191, p=.004, $\eta^{2}=0.173, 95\% \eta^{2}\mathrm{CI}[0.035, 0.328]$) and a significant direction by coherence interaction (F(4,176)=7.109, p<0.001, $\eta^{2}=0.139, 95\% \eta^{2}\mathrm{CI}[0.055, 0.203]$). The main effect of medication status (F(1,44)=0.618, p=0.436, $\eta^{2}=.014, 95\% \eta^{2}\mathrm{CI}[0.00, 0.112]$), the medication status by coherence interaction (F(2.977,130.993)=1.257, p=0.292, $\eta^{2}=.028, 95\% \eta^{2}\mathrm{CI}[0.00, 0.070]$), and the three-way interaction (F(4,176)=0.658, p=0.622, $\eta^{2}=0.015, 95\% \eta^{2}\mathrm{CI}[0.00, 0.032]$) were non-significant. Post-hoc tests of simple effects indicated that the unmedicated group (M=0.690, SD=0.140,) had significantly greater accuracy than the medicated group (M= 0.593, SD=0.139) in the same condition (p=.005, *d*=0.39, 95% dCI [0.12, 0.65]), but not the opposite condition. In fact, the medicated group (M = 0.862, SD=0.100) had significantly higher accuracy than the unmedicated group (M =0.807, SD=0.098) in the opposite condition (p=0.072, *d*=-0.30, 95% dCI [-0.56, -0.03]).

Two-way ANOVAS compared facilitation and suppression effects by medication status and coherence (Supplementary Figure 2-B). For facilitation, there was a significant main effect of coherence, F(2.996,131.832)=3.796, p=0.012, $\eta^{2}=0.079, 95\% \eta^{2}\mathrm{CI}[0.010, 0.145]$. The main effect of medication status (F(1,44)=2.922, p=0.094, $\eta^{2}=0.062, 95\% \eta^{2}\mathrm{CI}[0.00, 0.198]$) and the medication status by coherence interaction (F(2.996,131.832)=0.429, p=0.732, $\eta^{2}<0.01, 95\% \eta^{2}\mathrm{CI}[0.00, 0.031]$) were non-significant. For suppression, there was a significant main effect of medication status, F(1,44)=7.524, p=.009, $\eta^{2}=0.146, 95\% \eta^{2}\mathrm{CI}[0.022, 0.300]$. The main effect of coherence (F(3.389,149.109)=2.34, p=0.068, $\eta^{2}=0.05, 95\% \eta^{2}\mathrm{CI}[0.00, 0.100]$) and the medication status by coherence interaction (F(3.389,149.109)=0.526, p=0.687, $\eta^{2}=0.012, 95\% \eta^{2}\mathrm{CI}[0.00, 0.032]$) were non-significant.

## Exploratory Analyses

We ran a one-way ANOVA to test for a significant difference in average CSS in medicated depressed participants (N=19, M=0.17, SD=0.15) versus healthy controls (M=0.14, SD=0.15) and found no significant difference (F(1,45) = 0.292, p=0591, $\eta^{2}=0.006, 95\% \eta^{2}\mathrm{CI}[0.00, .116]$). We ran a second one-way ANOVA to check for a significant difference in average Same accuracy in medicated depressed participants (N=19, M=0.59, SD=0.18) versus healthy controls (N=28, M=0.60, SD=0.15) and found no significant difference (F(1,45)=0.045, p=0.833, $\eta^{2}=0.01, 95\% \eta^{2}\mathrm{CI}[0.00, 0.076]$).
