# Supplementary figures and images for: Reduced visual context effects in global motion processing in depression
Source: PLoS One. 2023 Sep 13;18(9):e0291513. doi: 10.1371/journal.pone.0291513 (PMC10499266; doi:10.1371/journal.pone.0291513)

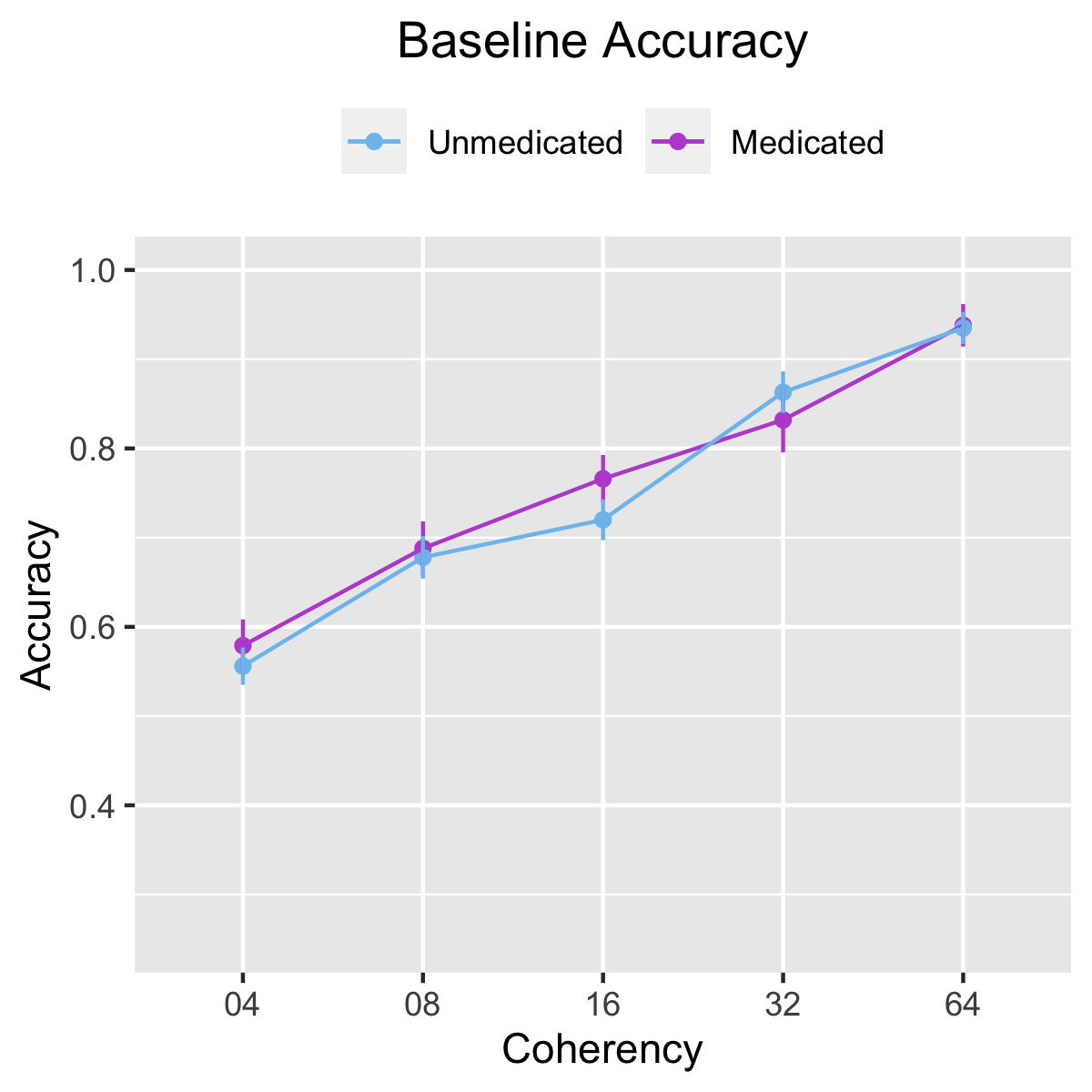

Supplement: S1 Fig — This figure depicts accuracy among medicated vs. unmedicated participants within the depressed group across all coherence levels. (TIF) [file pone.0291513.s002.tif]

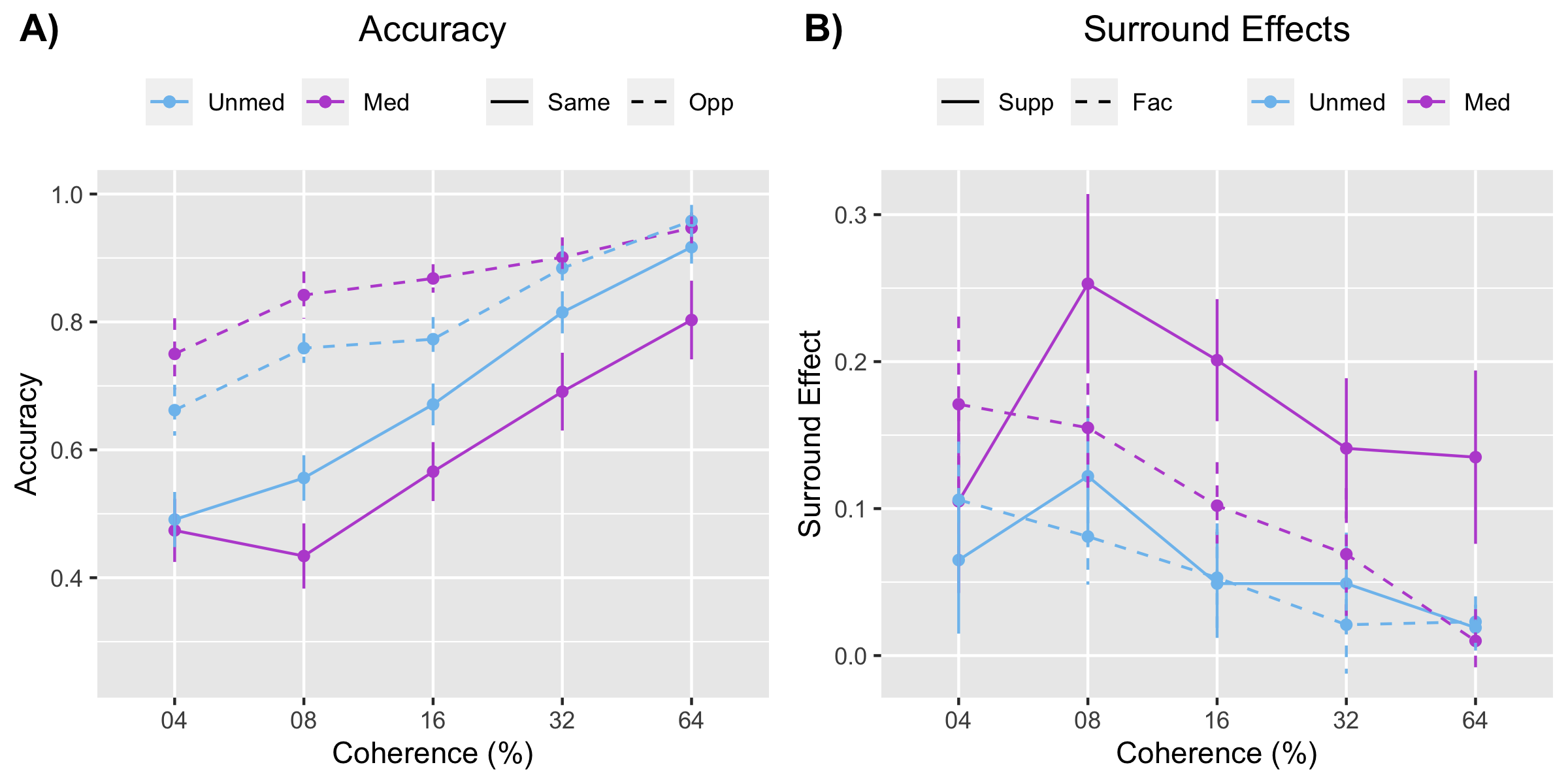

Supplement: S2 Fig — This figure depicts accuracy in the same and opposite conditions (Part A, left) and suppression and facilitation effects (Part B, right) among medicated vs. unmedicated participants within the depressed group across all coherence levels. (TIF) [file pone.0291513.s003.tif]
